# The mediating role of lifestyle in the relationship between shift work, obesity and diabetes

**DOI:** 10.1007/s00420-021-01662-6

**Published:** 2021-03-11

**Authors:** Gerben Hulsegge, Karin I. Proper, Bette Loef, Heleen Paagman, Johannes R. Anema, Willem van Mechelen

**Affiliations:** 1 Department of Public and Occupational Health, Amsterdam Public Health Research Institute, Amsterdam UMC, Vrije Universiteit Amsterdam, Van der Boechorststraat 7, 1081 BT Amsterdam, The Netherlands; 2 The Netherlands Organization for Applied Scientific Research, TNO, Schipholweg 77‑89, 2316 ZL Leiden, The Netherlands; 3 Centre for Nutrition, Prevention and Health Services, National Institute for Public Health and the Environment, Antonie van Leeuwenhoeklaan 9, 3721 MA Bilthoven, The Netherlands; 4 Department Research and Business Development, HumanTotalCare, Zwarte Woud 10, 3524 SJ Utrecht, The Netherlands; 5 Human Movement and Nutrition Sciences, Faculty of Health and Behavioural Sciences, University of Queensland, Brisbane, Australia; 6 Division of Exercise Science and Sports Medicine (ESSM), Department of Human Biology, Faculty of Health Sciences, University of Cape Town, Cape Town, South Africa; 7 School of Public Health, Physiotherapy and Population Sciences, University College Dublin, Dublin, Ireland

**Keywords:** Night work, Rotating shift system, Overweight, Type II diabetes, Diet, Sleep

## Abstract

**Purpose:**

Shift work has been related to obesity and diabetes, but the potential mediating role of lifestyle is yet unknown. Our aim was to investigate this mediating role of physical activity, diet, smoking, and sleep quality in the relationships between shift work, and obesity and diabetes.

**Methods:**

In this cross-sectional study, 3188 shift workers and 6395 non-shift workers participated between 2013 and 2018 in periodical occupational health checks. Weight and height were objectively measured to calculate obesity (BMI ≥ 30 kg/m^2^). Diabetes status, physical activity, diet, smoking, and sleep quality were assessed using standardized questionnaires. Structural equation models adjusted for relevant confounders were used to analyze the mediating role of lifestyle in the relationships between shift work, and obesity and diabetes.

**Results:**

Shift workers were more often obese (OR: 1.37, 95% CI 1.16–1.61) and reported more often to have diabetes (OR:1.35, 95% CI 1.003–1.11) than non-shift workers. Shift workers had lower physical activity levels, ate fruit and vegetables less often, smoked more often, and had poorer sleep quality (*p* < 0.05). Mediation analysis revealed that shift workers had a higher odds of obesity (OR: 1.07, 95% CI 1.01–1.15) and diabetes (OR: 1.13, 95% CI 1.02–1.27) mediated by poorer sleep quality. Lower physical activity levels (OR: 1.11, 95% CI 1.05–1.19) and lower intake of fruit and vegetables (OR: 1.04, 95% CI 1.01–1.15) were also mediators in the relationship between shift work and obesity, but not in the relationship between shift work and diabetes (*p* ≥ 0.05).

**Conclusion:**

These results imply that interventions targeting diet, physical activity and in particular sleep problems specifically developed for shift workers could potentially reduce the adverse health effects of shift work.

## Introduction

To serve the economic and societal demands of the 24/7 economy, it is necessary for a large part of the workforce to work around the clock. Shift work that includes night work has been recognized as one of the most prevalent occupational risk factors, affecting about 15–20% of the workforce in Europe and the U.S. (Parent‐Thirion et al. 2016; U.S. Bureau of Labor Statistics 2018). The high proportion of shift workers is a growing public health concern, because shift work increases the risk of chronic conditions, such as obesity and diabetes (Gan et al. 2015; Nea et al. 2015; Proper et al. 2016; van Drongelen et al. 2011). To develop preventive measures that mitigate the adverse health effects of shift work, it is necessary to understand the mediating pathways linking shift work to obesity and diabetes.

Several pathways have been proposed that link shift work to obesity and diabetes, including circadian disruption and stress related to disrupted hormonal and metabolic functions (James et al. 2017; Wang et al. 2011). A higher prevalence of unhealthy behaviors has also been hypothesized as an underlying pathway of the adverse health effects of shift work, as shift work-induced circadian misalignment affects the timing of exercise, eating, and sleep (Nea et al. 2015; Souza et al. 2019; Wang et al. 2011). Previous systematic reviews concluded that shift workers have poorer sleep quality and dietary patterns compared to non-shift workers (Amani and Gill 2013; Linton et al. 2015; Souza et al. 2019), although this may differ by sex and educational level (Kelly et al. 2020). There are also indications from a few observational studies that shift workers are less physically active and that they smoke more often than non-shift workers (Loprinzi 2015; Nabe-Nielsen et al. 2011; Trinkoff and Storr 1998; van Amelsvoort et al. 2006; Vandelanotte et al. 2015). One study found that middle-aged shift workers were in particular less physically active (Kelly et al. 2020). As these unhealthy lifestyle behaviors are also in shift workers related to obesity and diabetes (Cappuccio et al. 2010; Chiolero et al. 2008; Filozof et al. 2004; Jeon et al. 2007; O’Brien et al. 2020; Patel and Hu 2008; Willi et al. 2007), they may mediate the relationships between shift work, and obesity and diabetes. However, studies investigating the mediating role of lifestyle in the relationship between shift work and obesity and diabetes are currently lacking (O’Brien et al. 2020). Mediation analysis is particularly helpful to explore the explanatory role of lifestyle by estimating the magnitude of the mediating effect of lifestyle in the relationships between shift work and health outcomes. Therefore, our aim was to investigate the mediating role of physical activity, diet, smoking, and sleep quality in the relationships between shift work, and obesity and diabetes.

## Methods

### Population

A nationwide Dutch occupational health care service has continuously gathered data about work and health using standardized questionnaires and physical examinations, as part of their standard voluntarily occupational health checks among workers. Cross-sectional data from 22 industrial production companies were used for the present study. In total, 16,285 workers participated in a health check between 2013 and 2018, enriched with data on type of shift schedule from company records. We excluded participants without data on shift work (*n* = 1223), those with a specific irregular shift schedule in which relatively few participants work (e.g. 2-shift workers, off-shore workers) (*N* = 1001), former shift workers (*N* = 312), or those with missing data on obesity and diabetes (*N* = 3264), lifestyle (*N* = 660) or covariates (*N* = 242). This resulted in a study population of 6395 non-shift workers and 3188 shift workers. The included workers worked in manufacturing companies, for example, as manufacturing and assembly workers, operators, team/shift leaders, and office worker. The Medical Ethics Committee of the VU University Medical Center Amsterdam approved the study.

### Shift work

The Human Resources department of most companies provided for each worker information on the type of shift work, i.e. 3-shift work, 4-shift work, 5-shift work, and non-shift work. In general, the 3-shift schedule consisted of a slow-forward rotating schedule of a week morning shift (e.g. from 6:00 to 14:00) followed by a week afternoon (e.g. from 14:00 to 22:00) and night shifts (e.g. from 22:00 to 6:00), with two days off during the weekend. The 5-shift schedule consisted of a fast-forward rotating schedule of two morning shifts, two afternoon shifts, and two night shifts, followed by three or four days off work. The 4-shift schedule alternated also between two or three morning, afternoon and night shifts. The 3-, 4-, and 5-shift schedules were combined into one category, as very few participants had a 3- or 4-shift schedule and all these shift schedules rotated between morning, afternoon, and night shifts.

### Outcomes

Body weight and height were objectively measured by trained assistants/nurses. Obesity was defined as a body mass index ≥ 30 kg/m^2^. Diabetes status was based on self-report with the question: “Do you have diabetes”, with the answer options “yes” and “no”.

### Lifestyle

Lifestyle factors were measured using a standardized questionnaire. Participants were asked for how many days of the week they had performed on average leisure time moderate-to-vigorous intensity physical activity (LTPA), for at least 30 min per day. In line with the physical activity guidelines, that recommend to meet at least 150 min/week of LTPA (Piercy et al. 2018; Weggemans et al. 2018), LTPA was dichotomized into < 5 days/week and ≥ 5 days/week. Diet was measured using four questions. Participants were asked how much per day and on how many days of the week they usually ate fruit and vegetables. We standardized fruit and vegetable intake as the number of servings per day, and 77 g of vegetables was considered to be one serving, as calculated in a previous study (He et al. 2006). We dichotomized fruit and vegetable intake based on the median intake as: low intake (< 3 portions/day), and; high intake (≥ 3 portions/day). This cut-off is in line with the meta-analysis of He et al. (2006), which showed that intake of more than three fruits and vegetables per day was associated with lower risk of cardiovascular disease.. Smoking was asked for with a single question and dichotomized into current smoker and non-smoker. Sleep was measured by calculating the average of the scores on the following four statements (Cronbach’s α: 0.88): Last month I (1) had the feeling not to be able to close an eye; (2) slept restless; (3) had difficulty falling asleep; and (4) woke up tired. Answer options ranged from never (score ‘0′) to daily (score ‘4′) on a 5-point Likert scale. This variable was dichotomized at the 75 percentile into good (score < 2.5) and poor (score ≥ 2.5) sleep quality.

### Covariates

Age (continuous), gender (men vs. women), education (intermediate secondary education or less vs. intermediate vocational or higher secondary education vs. higher vocational education or university), children living at home (yes vs. no/not applicable) and working hours/week were self-reported. Type of work tasks was assessed using the question “What type of occupation/work do you have?”, with three response options: mainly physically demanding work tasks, mainly mentally demanding work tasks, or a combination of the two.

### Data analysis

**Fig. 1:**
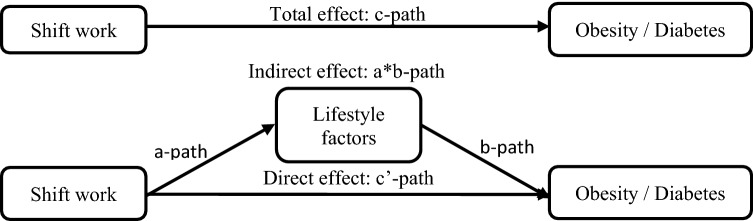
Visualization of the multiple-mediation model

Baseline characteristics of non-shift and shift workers were compared using the independent samples t-test and Chi-square test. as means (SD) and number (percentage). As some companies did not offer anthropometric measurements to their employees, obesity was not measured in 23% of the population. Characteristics of the population with complete data on obesity (77%) were also calculated. The relationships between shift work, and obesity and diabetes were estimated using logistic regression analyses. Logistic regression Structural Equation Models (SEMs) were used to analyze the mediating role of lifestyle in the relationships between shift work, and obesity and diabetes. Figure 1 shows the mediation model. The total effects (c-paths) of shift work on obesity and diabetes are shown in the upper part of the figure and were adjusted for the confounders age, gender, education, children living at home, working hours/week, and type of work tasks. The lower part of Fig. 1 shows the mediation model of the indirect effects of lifestyle (a- and b-paths), as well as the direct effects of shift work (c’-paths) on obesity and diabetes, which were adjusted for all potential mediators and confounders. The indirect effect of each lifestyle factor (mediator) was calculated as the product of the a- and b-paths in the multiple-mediation models, including all potential mediators and confounders in one model (Sobel 1982). The 95% confidence intervals around the indirect effects were calculated based on 5000 bootstrap resamples. We also performed sensitivity analyses in which we excluded women and those with a 2-, 3- or 4-shift system, because there were only few of such individuals. All analyzes were performed using Stata/SE, version 14.1 (StataCorp LLC, College Station, Texas), and a two-sided *p*-value < 0.05 was considered statistically significant.

## Results

### Study population

Shift and non-shift workers were on average 46 years old (SD: 11) (*P* = 0.20) (Table 1) and most of the participants were male. Compared to non-shift workers, shift workers were more often low educated (9% vs 27%) and had more physically demanding jobs (13% vs 33%) (*P* < 0.01). Most shift workers worked in a 5-shift schedule (95%), and did so for 19 years on average.

**Table 1:** Characteristics of the study population

	Non-shift worker *N* = 6395	Shift worker *N* = 3188	*P*-value for difference between non-shift and shift workers
Demographics			
Age (years)	45.7 ± 10.6	46.0 ± 10.9	*P* = 0.20
Gender (male)	5556 (79%)	3085 (97%)	*P* < 0.01
Educational level			*P* < 0.01
Low	602 (9%)	875 (27%)	
Intermediate	2212 (35%)	2140 (67%)	
High	3581 (56%)	173 (5%)	
Living with children	3631 (57%)	1719 (54%)	*P* < 0.01
Occupational			
Type of job			*P* < 0.01
Physical	828 (13%)	1036 (33%)	
Mental	4860 (76%)	507 (16%)	
Combination	707 (11%)	1645 (52%)	
Years worked in shifts	NA	19.2 ± 10.4	NA
Working hours per week	39.3 ± 6.8	34.6 ± 4.3	*P* < 0.01
Type of shift worker			NA
3-shift worker	NA	132 (4%)	
4-shift worker	NA	36 (1%)	
5-shift worker	NA	3020 (95%)	
Anthropometry			
BMI*	26.0 ± 3.8	27.2 ± 3.9	*P* < 0.01
Overweight/obesity*	2673 (55%)	1794 (70%)	*P* < 0.01
Obesity^a^	732 (15%)	603 (24%)	*P* < 0.01
Diabetes	166 (2.6%)	151 (4.7%)	*P* < 0.01
Lifestyle			
MVPA (30 min for < 5 day/week)	3701 (58%)	1850 (58%)	*P* = 0.90
Fruit & vegetables (< 3 portions)	2986 (47%)	1983 (62%)	*P* < 0.01
Smoking (yes)	966 (15%)	1034 (32%)	*P* < 0.01
Sleep quality (poor)	1644 (26%)	1160 (36%)	*P* < 0.01

Values represent means ± standard deviations, numbers and (percentages)

yBMI: body mass index; MVPA: moderate-to-vigorous physical activity; NA: not applicable

^a^Data available for 4848 non-shift workers and 2569 shift workers

Shift workers had more often obesity (24%) and diabetes (5%) than non-shift workers (15% and 3%, respectively) (*P* < 0.01) (Table 1). Shift workers also had a less healthy lifestyle. Shift workers had more often a low intake of fruit and vegetables (62% vs. 47%) and a poorer sleep quality (36% vs. 26%), and they smoked more often (32% vs. 15%) than non-shift workers (*P* < 0.01). There was no difference in physical activity level between shift and non-shift workers (both 58%) (*P* = 0.90). Characteristics of the subsample with complete data on obesity (*N* = 7,417, 77%) did not substantially differ from the total population (data not shown).

### Mediation model obesity

The total effect (c-path) of shift work on obesity in the multiple-adjusted logistic regression model was 1.37 (95% CI 1.16–1.61), meaning that shift workers had a 1.37 higher odds of being obese compared to non-shift workers (Table 2). The OR of the direct effect (c’-path) of shift work on obesity in the model with all mediators included was 1.35 (95% CI 1.15–1.60). Compared to non-shift workers, shift workers were more often physically inactive, ate fruit and vegetables less often, smoked more often, and had more often poor sleep quality (a-paths). Shift and non-shift workers who were physically inactive, had poorer sleep quality, and those who ate less often fruit and vegetables had an increased odds of obesity, whereas those who smoked had less often obesity compared to non-smokers (b-paths). The indirect effects shown in Table 2 indicate the extent to which the relationship between shift work and obesity was mediated by the poorer lifestyle behaviors among shift workers. Shift workers had a 1.11 times higher odds of obesity via being more often physically inactive (95% CI 1.05–1.19) compared to non-shift workers. The higher odds of obesity among shift workers was also mediated via a lower intake of fruits and vegetables (OR: 1.04, 95% CI 1.01–1.09) and poorer sleep quality (OR: 1.07, 95% CI 1.01–1.15). In contrast, shift workers had a lower odds of obesity via a higher prevalence of smoking compared to non-shift workers (OR: 0.79, 95% CI 0.70–0.87).

**Table 2:** Path odds ratios (95% confidence intervals) of lifestyle factors on the relationship between shift work and obesity

	Total effect c-path (shift work > obesity)	Direct effect c’-path (shift work > obesity)	a-paths (shift work > lifestyle factor)	b-paths (lifestyle factor > obesity)	Indirect effects a*b-path (shift work > lifestyle factor > obesity)
Shift work	**1.37 (1.16–1.61)**	**1.35 (1.15–1.60)**			
Physical inactivity			**1.36 (1.19–1.55)**	**1.41 (1.24–1.60)**	**1.11 (1.05–1.19)**
Low intake of fruit & vegetables			**1.29 (1.13–1.47)**	**1.17 (1.03–1.32)**	**1.04 (1.01–1.09)**
Smoking			**1.63 (1.40–1.90)**	**0.62 (0.53–0.73)**	**0.79 (0.70–0.87)**
Poor sleep quality			**1.51 (1.31–1.74)**	**1.18 (1.04–1.35)**	**1.07 (1.01–1.15)**

Structural equation model adjusted for age, gender, education, children living at home, working hours/week, and type of work tasks. Boldface indicates statistical significance (*p* < 0.05)

### Mediation model diabetes

Shift workers had a higher odds of diabetes compared to non-shift workers (OR: 1.35, 95% CI 1.003–1.81) (Table 3). The direct effect was 1.31 (95% CI 0.97–1.76), after inclusion of the mediators in the model. Physical inactivity, low fruit and vegetable intake, and smoking were not related to diabetes (*p* ≥ 0.05), while poor sleep quality was significantly related to a higher odds of diabetes (OR: 1.34, 95% CI 1.06–1.71) (b-paths). Poor sleep quality was also the only significant mediator in the relationship between shift work and diabetes, with shift workers having a 1.13 times higher odds of diabetes than non-shift workers via poorer sleep quality (95% CI 1.02–1.27).

**Table 3:** Path odds ratios (95% confidence intervals) of lifestyle factors on the relationship between shift work and diabetes

	Total effect c-path (shift work > diabetes)	Direct effect c’-path (shift work > diabetes)	a-paths (shift work > lifestyle factor)	b-paths (lifestyle factor > diabetes)	Indirect effects a*b-path (shift work > lifestyle factor > diabetes)
Shift work	**1.35 (1.003–1.81)**	1.31 (0.97–1.76)			
Physical inactivity			**1.39 (1.24–1.56)**	1.03 (0.81–1.29)	1.01 (0.93–2.41)
Low intake of fruit & vegetables			**1.25 (1.12–1.41)**	1.10 (0.87–1.39)	1.02 (0.97–1.09)
Smoking			**1.59 (1.39–1.82)**	0.84 (0.63–1.13)	0.92 (0.79–1.05)
Poor sleep			**1.51 (1.33–1.70)**	**1.34 (1.06–1.71)**	**1.13 (1.02–1.27)**

Structural equation model adjusted for age, gender, education, children living at home, working hours/week, and type of work tasks. Boldface indicates statistical significance (*p* < 0.05)

### Sensitivity analyses

Exclusion of women and those working in a 2-, 3- or 4-shift system provided virtually the same results as the main analyses (data not shown).

## Discussion

We aimed to study the mediating role of lifestyle factors in the relationships between shift work, and obesity and diabetes. The relationship between shift work and obesity was mediated by physical inactivity, poor diet, and poor sleep quality. In contrast, shift work was related to a lower odds of obesity via smoking. The higher odds of diabetes among shift workers was mediated by poorer sleep quality, but not via any of the other lifestyle factors.

The finding of a higher prevalence of obesity among shift workers compared to non-shift workers is in line with findings of previous review studies (Proper et al. 2016; van Drongelen et al. 2011). What this study adds to previous research is the observation that among production workers the relationship between shift work and obesity was mediated by being more often physically inactive, eating fewer fruits and vegetables, and having a poorer sleep quality. Although mediation analyses regarding the relationship between shift work and diabetes/obesity have, to our knowledge, not been performed, previous research indicated that shift work is related to unhealthy lifestyle behaviors. Shift workers have been shown to have more often difficulties with initiating, maintaining and consolidating sleep, and consequently have poorer sleep quality, compared to non-shift workers (Akerstedt 2003; Linton et al. 2015; Niu et al. 2011). Previous systematic reviews also indicated that shift workers have poorer dietary behaviors (Amani and Gill 2013; Souza et al. 2019), but results for the intake of fruit and vegetables were mixed across studies. For example, the review of Amani and Gill (2013) concluded that in four studies shift workers consumed less fruit and vegetables than non-shift workers, while in three studies shift workers had a higher intake of fruit and vegetables. Studies on the relationship between shift work and physical activity were giving also mixed results, with some studies showing shift workers to be less physically active than non-shift workers (Loprinzi 2015; Nabe-Nielsen et al. 2011; Vandelanotte et al. 2015), while other studies did not find differences in physical activity levels between shift and non-shift workers (Atkinson et al. 2008; Loef et al. 2017; Thomas and Power 2010). The present study strengthens the evidence that shift workers in the production industry are less moderate-to-vigorous physically active during leisure time and that they eat less fruit and vegetables than non-shift workers in the production industry. Altogether, physical inactivity, low intake of fruits and vegetables, and poor sleep quality are likely important mediators, that explain the relationship between shift work and obesity. However, longitudinal studies with more detailed, preferably objective, measurements of physical activity and diet are necessary to confirm our findings before strong conclusions can be drawn.

In contrast to physical activity, diet, and sleep quality, smoking was not a mediator in the adverse relationship between shift work and obesity. In line with the few available studies, the present study showed that shift workers smoked more often than non-shift workers (Trinkoff and Storr 1998; van Amelsvoort et al. 2006). However, smoking was related to a lower odds of being obese, and, thereby, smoking seemed to ‘protect’ shift workers against obesity. This is in line with our previous study, that showed that the relationship between shift work and overweight was weaker in smokers than non-smokers (Hulsegge et al. 2020). This is supported by systematic reviews showing that smokers have lower body weight than non-smokers, and that people gain weight after quitting smoking (Aubin et al. 2012; Chiolero et al. 2008; Tian et al. 2015). Although underlying mechanisms remain poorly understood, this might be because smoking and, more specifically nicotine, increases energy expenditure and could reduce appetite (Chiolero et al. 2008). Thus, the results of the present study indicate that the higher odds of obesity among shift workers is in part mitigated by the higher prevalence of smoking among shift workers, compared to non-shift workers. For the purpose of reducing the high obesity prevalence among shift workers, this study highlights the importance of increasing physical activity, improving diet and sleep quality among shift workers. From a public health perspective, the high prevalence of smoking among shift workers is worrying due to the high risk of developing chronic diseases, such as cardiovascular diseases.

The present study observed sleep quality, but none of the other lifestyle factors, to mediate the relationship between shift work and diabetes. Disturbed sleep among shift workers is often caused by circadian misalignment, including inappropriately timed sleep, wake and feeding rhythms, increases in neuroendocrine stress systems, and elevated stress responses (Kecklund and Axelsson 2016). This in turn, may lead to glucose intolerance and insulin resistance, increasing the risk of type 2 diabetes (Spiegel 2008). This is in line with a meta-analysis that showed that people who had difficulties initiating and maintaining sleep had a 1.57 and 1.84 times higher risk to develop type 2 diabetes, respectively (Cappuccio et al. 2010). In contrast to expectations, the other lifestyle factors did not mediate the relationship between shift work and diabetes, while physical inactivity, poor diet and smoking have previously been found to be significant risk factors for the development of type 2 diabetes (Jeon et al. 2007; Psaltopoulou et al. 2010; Willi et al. 2007). The reasons why in our study these lifestyle factors mediate the relationship between shift work and obesity, but not the relationship between shift work and diabetes, are unclear. One reason might be that the relationships between physical activity and smoking, and diabetes may have been underestimated in our study, because diabetes cases without a formal diagnosis could not been identified using self-reported diabetes status. Another reason might be that poor diet and physical inactivity are more distal in the causal chain of events in the development of diabetes compared to obesity (i.e. diet and physical activity have a less direct effect on diabetes than on obesity), making it more difficult to prove the mediating role of diet and physical activity for diabetes. Thus, the present study provides indications for a mediating role of sleep quality in the relationship between shift work and diabetes, but further exploration of the mediating role of other lifestyle factors is needed.

The strength of the present study is the use of a large homogeneous group, which minimizes residual confounding related to differences across companies, such as the organizational culture of companies. This implicates that lifestyle factors play a moderating role in different types of companies, but the role of lifestyle factors—and in particular diet and physical activity- in explaining the health effects of shift work may differ across companies due to environmental differences. For example, a previous study showed that shift workers with access to workplace vending were more likely to regularly drink soft drinks, and those with adequate break times at work were more likely to be physically active (Kelly et al. 2020). Another strength is that shift work schedule and history of shift work were determined using objective registry data, and multiple relevant behavioral mediators were studied. The cross-sectional design is a limitation of the present study. Reverse causality cannot be ruled out as obesity and diabetes may influence lifestyle behaviors, and it remains unclear whether the unhealthy lifestyle behaviors were the cause, or the consequence of obesity and diabetes. Body weight and body height were not measured in 23% of the population. We expect this to have had little impact on the results, because missing data is neither due to self-selection bias, nor to non-response bias of invitees, but due to the companies’ choice not to offer medical examinations to their employees. This hypothesis is supported by our finding of similar characteristics among the subpopulation with complete data on obesity and the total population. Physical activity and diet were measured using relatively general measures. Unfortunately, we had no further information on other important aspects of an unhealthy diet, such as intake of fast foods, sweets, and salty snacks. As our measurements did not capture the full spectrum of physical activity and dietary patterns, the mediating role of these lifestyle factors may have been underestimated. Our classification of healthy and unhealthy diet based on the median intake of fruit and vegetables overestimates the proportion of participants with a healthy diet, as dietary guidelines advice higher intakes (U.S. Department of Health and Human Services and U.S. Department of Agriculture 2015). This may also have contributed to an underestimation of the mediating role of fruit and vegetables. More research is recommended to investigate which aspects of diet and physical activity explain the adverse health effect of shift work to better target prevention. Generalization of the results is limited to male blue-collar workers with rotating shift schedules that include night shifts. This is an important group of workers as this group represents a large part of the total shift work population with relatively unhealthy lifestyle behaviors and a high risk of developing chronic diseases (McMenamin 2007). The healthy worker effect is a common methodological problem in shift work research, because workers who have obesity or diabetes may be less likely to start shift work and may be more likely to leave shift work, compared to healthy workers (Nabe-Nielsen et al. 2008). Although we observed relationships between shift work, and obesity and diabetes, the healthy worker effect may have underestimated these relationships.

To conclude, shift workers had a higher odds of obesity and diabetes and a less healthy lifestyle than non-shift workers. The higher prevalence of obesity among shift workers was mediated by physical inactivity, eating fewer portions of fruits and vegetables, and poorer sleep quality. The relationship between shift work and diabetes was mediated by poorer sleep quality, but not by any of the other lifestyle factors. These results imply that increasing physical activity levels and improving diet and sleep quality should be priorities in the prevention of obesity and diabetes among shift workers.

## Data Availability

Due to ethical restrictions related to participant consent and high sensitivity of the company data, all relevant data are under the conditions of HumanTotalCare available upon request to the responsible senior manager Research & Business Development at HumanTotalCare: Heleen Paagman (email: heleen.paagman@arboned.nl).
